# Fabrication of curcumin-loaded electrospun nanofiberous polyurethanes with anti-bacterial activity

**DOI:** 10.1007/s40204-017-0079-5

**Published:** 2017-12-01

**Authors:** A. Shababdoust, M. Ehsani, P. Shokrollahi, M. Zandi

**Affiliations:** 1 Plastics Department, Iran Polymer and Petrochemical Institute, Tehran, P.O. Box 14965/115, Iran; 2 Biomaterials Department, Iran Polymer and Petrochemical Institute, Tehran, P.O. Box 14965/115, Iran

**Keywords:** Polyurethane, Electrospinning, Curcumin release, Anti-bacterial activity

## Abstract

**Abstract:**

Two series of polyurethane (PU), based on polycaprolactone (PCL) as soft segments with two different molecular weights (2000 and 530 Da), and hexamethylene diisocyanate (HDI) and 1,4-butandiol (BDO) as hard segments were synthesized to fabricate curcumin-loaded electrospun nanofibrous PCL-based PU substrate. Chemical structures of the synthesized PUs were characterized by FTIR and NMR spectroscopy techniques. The thermal properties were analyzed by differential scanning calorimetry (DSC) and surface hydrophilicity was studied by static contact angle and bulk hydrophilicity was evaluated by water uptake test. Thereafter, bead-free PU nanofiberous substrate containing curcumin was fabricated by electrospinning and morphology of the mats was observed by scanning electron microscopy (SEM). Mechanical properties of the electrospun mats in comparison with polymeric films were assessed by a universal test machine. The in vitro release of curcumin was studied by UV–Vis spectroscopy. The optical density of the bacterial solutions was used to evaluate the antibacterial activity of the curcumin-loaded nanofibrous mats against Escherichia coli (E-coli ATCC: 25922). The results showed that curcumin-loaded PU synthesized by PCL with molecular weight of 2000 Da displayed better mechanical properties as well as better antibacterial properties in wound dressing application.

**Graphical abstract:**

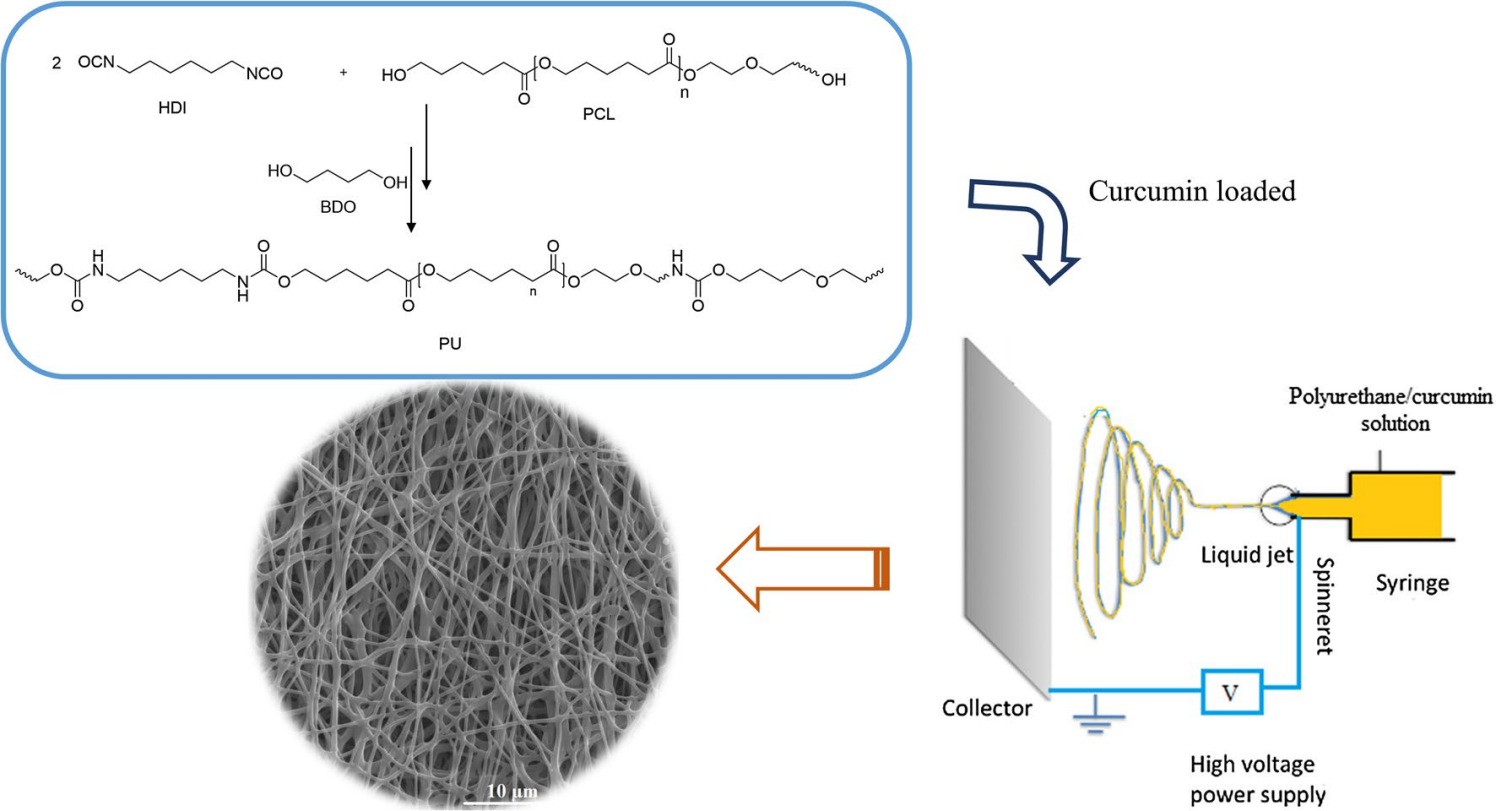

## Introduction

Polyurethane refers to a class of polymers containing urethane bond (–NHCOO-group) in the main chain (Gogolewski 1989) that are made by reaction of diisocyanate, a polyol and a small diol and or diamine molecule used as chain extenders (Krol 2007). Due to various structures of the original reactants and the existence of soft and hard segments and the resulting interactions between them, polyurethanes show a wide range of physical and mechanical properties, which make them unique as polymeric materials in many applications such as resins (Pathak et al. 2009), adhesives (Malucelli et al. 2005), foams (Jain and Pradeep 2005), waterborne paints (Yang et al. 2004) and rubbers (Varghese et al. 2004). PUs are used in biomedical applications such as artificial heart valve (Yu et al. 1989), blood vessel (Pennings et al. 1990), joints (Scholes et al. 2007) as well as controlled release system (Sivak et al. 2008) and wound dressing (Unnithan et al. 2014).

Curcumin, known as (bis-1,7-[4-hydroxy-3-methoxyphenyl]-hepta-1,6-dione), is derived from curcuma longa (Goel et al. 2008; Himesh et al. 2011) and the yellow pigment known as turmeric (Gunes et al. 2013). Curcumin has many medical applications because of outstanding properties such as anti-inflammatory (Chandra and Gupta 1972; Huei-Chen et al. 1992), antioxidant (Erenoğlu et al. 2011), burn wound healing (Kulac et al. 2013), antifungal (Martins et al. 2009) and antibacterial (Gunes et al. 2013) agent.

Electrospinning is a method of fiber fabrication with diameters ranging from micro-meter to nano-scale by accelerating a jet of charged polymer solution in an electric field (Natu et al. 2011). These fibers can be produced from natural (Zhang et al. 2005) and synthesized polymers (Demir et al. 2002; Saeed et al. 2017) with different applications such as filteration (Gopal et al. 2006), catalyst (Patel et al. 2007) drug delivery (Zeng et al. 2003), tissue engineering (Li et al. 2002) and wound dressing (Khil et al. 2003). In some studies polyurethane has been used as carrier for curcumin (Abdollahi et al. 2015; Nagarajan et al. 2011; Souguir et al. 2013) and also in some studies curcumin is loaded into a synthesized polymer such as cellulose acetate (Suwantong et al. 2007), polyvinylalcohol (PVA) (Sun et al. 2013), zein (Brahatheeswaran et al. 2012), poly (lactic acid) (PLA) (Chen et al. 2010), poly(*dl*-lactic-*co*-glycolic) acid (PLGA) (Sampath et al. 2014), poly(ε-caprolactone) (PCL) (Merrell et al. 2009) and p(HEMA) (Merrell et al. 2009). Based on our knowledge, we may declare that electrospun nanofibres of polyurethane containing curcumin are not fabricated before.

In this study we have synthesized polyurethane, a biocompatible polymer as carrier for antibacterial drug by hexamethylene diisocyanate (HDI) as diisocyanate, polycaprolactone (PCL) as biocompatible polyol and butanediol (BDO) as chain extender. To control the mechanical properties as well as the drug loading content of the synthesized polymeric system, two different molecular weights of PCL (530 and 2000 Da) were introduced into the polymer backbone; with curcumin, an anti-bacterial and wound healing agent, as loading drug to fabricate curcumin-loaded polyurethane nanofibrous mat. Nanofibrous containing curcumin was fabricated by electrospinning process because of many advantages such as large surface area and superior physical properties. Following the fabrication of drug-loaded electrospun nanofibers, the anti-bacterial properties of the fabricated mats were investigated by comparing two systems based on PCL molecular weights.

## Experimental

### Materials

Polycaprolactone (PCL) (*M*_n_ 2000 and 530 Da) was purchased from Sigma–Aldrich; 1,6-hexamethylene diisocyanate (HDI), 1,4-butanediol (BDO), 1,2-dicholoethane, stannous octoate, curcumin (Cur) and 1,1,1,6,6,6-hexafluoroisopropanol (HFIP) were purchased from Merck Co. PCL and BDO were dried prior to use under vacuum at 80 °C for 24 h.

### Synthesis of polyurethanes

Polyurethanes were synthesized by a two-step polymerization method. Briefly, in the first step 1 mmol PCL (*M*_n_ 2000 and 530 Da, in separate procedures, referred to as PCL2000 and PCL530 hereafter) was transferred to a three-necked flask, and were added 2 mmol HDI with 1,2-dichloroethane and 0.1% wt stannous octoate. The mixture was heated for 4 h at 70 °C under dry nitrogen atmosphere. After 4 h, 1 mmol BDO was added into the reaction system and allowed to continue for 24 h under dry nitrogen atmosphere. The product was cooled down and then precipitated in *n*-hexane. The precipitate was dissolved in chloroform and re-precipitated in *n*-hexane to eliminate stannous octoate and possible oligomeric residues. Final product was dried under vacuum at 40 °C for 72 h. Table 1 shows molar ratio and hard segment content of the synthesized polyurethane.

**Table 1:** Sample coding, molar ratio and hard segment content of the synthesized polyurethane

Sample code	HDI (mmol)	PCL (Mw) (mmol)	BDO (mmol)	Hard segment content (%)
PU2000	2	1 (2000 Da)	1	17.5
PU530	2	1 (530 Da)	1	44.6

### Characterization

FTIR and ^1^H NMR spectroscopy were used to identify the chemical structures of the synthesized copolymers, trace the reaction and determine the polyols ratio in the final products. Infrared spectroscopy was performed on a Bruker instrument (Aquinox 55, Germany) in the range of 400–4000 cm^−1^. The ^1^H NMR spectrum was recorded in CDCl_3_ as a solvent using a 500-MHz Bruker spectrometer (DRX-500 Avance, Germany).

Thermal behavior of the samples was evaluated by a Mettler differential scanning calorimeter (DSC) (Star SW 10.00, Switzerland). The copolymers were heated to 180 °C and cooled down to − 80 °C and re-heated to 180 °C. Thermal transitions were extracted from the second heating run.

Mechanical properties of the copolymers were investigated by a Santam mechanical testing machine (STM-20, with a 200 N load cell, Iran), at a crosshead speed of 5 mm min^−1^, according to ASTM D 638. The tensile strength (*σ*_s_), Young’s moduli (*E*) and elongation-at-break (*ε*) of polymer films (30 mm × 10 mm × 0.3 mm) were measured. The reported data were the average data of three specimens.

Surface hydrophilicity was investigated by water contact angle method. The reported results were the average of three measurements using a Kruss G10 contact angle measuring system (Germany).

Bulk hydrophilicity was studied by water uptake of copolymers according to the reference (Skarja and Woodhouse 1998) and was defined as the difference of the wet mass (*w*_2_) and dry mass (*w*_1_) of the films:

1 $$ {\text{Water}}\,{\text{uptake}}\,(\% ) = \frac{{(w_{2} - w_{1} )}}{{w_{1} }} \times 100. $$

### Fabrication and characterization of curcumin-loaded electrospun nanofibers

For fabrication the bead-free electrospun mat of polyurethane and polyurethanes/curcumin, two separate solutions were prepared by (1) 20 wt% polyurethane, synthesized by PCL of *M*_n_ 2000 Da and (2) 30 wt% polyurethane synthesized by PCL of *M*_n_ 530 Da in HFIP. To achieve the polymeric solutions containing curcumin, 5% curcumin with respect to the PU content was added to the PU2000 and PU530 solutions and 10% curcumin with respect to the PU2000.

Electrospinning was performed using the following parameters: 20 kV applied voltage, 21 cm tip-to-collector distance, 0.5 mL h^−1^ solution flow rate and 1000 rpm collector rotation. The electrospun mats were dried under vacuum to eliminate the residual solvent for 72 h.

Morphology of the electrospun nanofibers were studied by scanning electron microscopy (SEM: VEGA 3SBH\\TESCAN Brno). The average fiber diameter was calculated by analysis of around 150 fibers in SEM images using the Image J software and the histogram of frequency/fiber diameter for each sample was obtained.

Mechanical properties of the electrospun nanofiber mats were investigated by a Santam mechanical testing machine (model STM-20, with a 10 N load cell, Iran) at a crosshead speed of 1 mm min^−1^. The tensile strength (*σ*_s_), Young’s moduli (*E*) and elongation-at-break (*ε*) of the 5% curcumin loaded electrospun polymer mats (30 mm × 10 mm × 0.3 mm) were measured. The reported data were the average data of three specimens.

### Curcumin release study

Because of low solubility of curcumin in water, its release was studied in a mixture of water and ethanol (70:30) as a release medium (Alexis et al. 2004; Zhu et al. 2015). So, 20–30 mg of 5% curcumin-loaded and 10–20 mg of 10% curcumin-loaded electrospun mats were placed into 15 mL release medium and transferred into the shaking incubator at 37 °C and 100 rpm.

At a regular time interval, the liquid media were replaced by aliquots of fresh media. Next, the sample solutions were analyzed at wavelength of 429 nm by a UV spectrophotometer. Accumulative drug release percent was calculated by using a calibration curve and it was plotted versus time. All measurements were performed three times and the results were average values of at least three sequencing tests.

### Invitro anti-bacterial properties

The optical density values of the bacterial solutions were used to evaluate the antibacterial activity of the curcumin-loaded nanofibrous mats against Escherichia coli (E-coli ATCC: 25922). The bacterial suspensions were prepared by 0.5 McFarland bacterial suspension method briefly outlined as follows: 10^8^ colony-forming units per milliliter (CFU mL^−1^) was diluted to 10^5^ (CFU mL^−1^) in DifcoTM nutrient broth solution. Curcumin-loaded electrospun mats, each 50 mg, were cut and after sterlization by UV-lamp and were placed in 5 mL of bacterial solution. The mixtures were cultured at 37 °C in a shaking incubator for 48 h. The turbidity of the medium, which represented the bacterial growth, was measured after 48 h using a spectrophotometer at 600 nm wavelength. The antibacterial efficiency of the curcumin-loaded nanofiber mats was then calculated from the following equation (Nguyen et al. 2011):

2 $$ {\text{Antibacterial}}\;{\text{efficiency}} \;\left( \% \right) = \left( {1 - \frac{{{\text{OD}}_{1} }}{{{\text{OD}}_{2} }}} \right) \times 100, $$

where OD_1_ and OD_2_ are the optical densities of the bacteria in the medium and the bacteria in solutions containing different nanofiber mats, respectively, for 48 h.

## Results and discussion

### Polyurethane synthesis

**Fig. 1:**
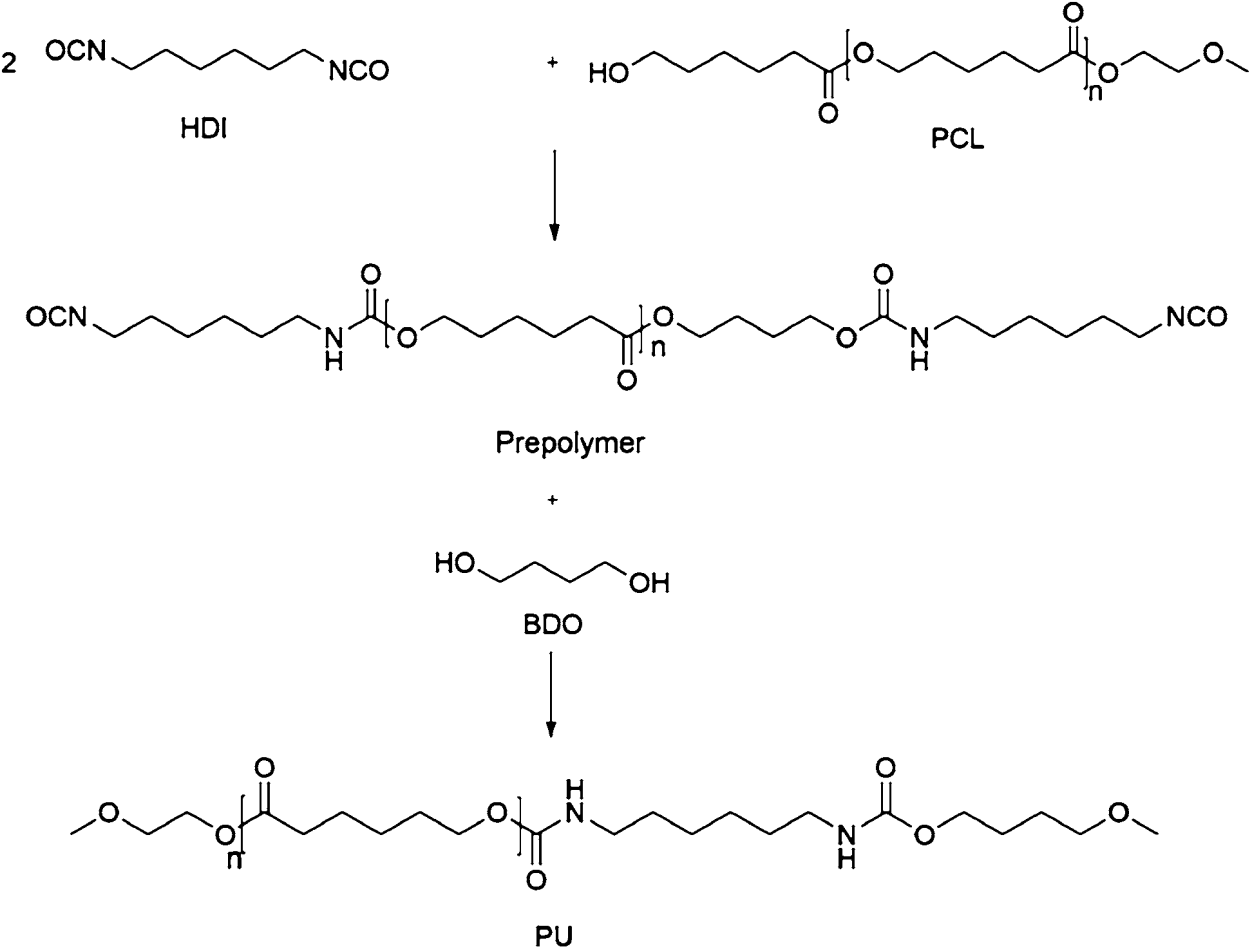
Schematic presentation of a two-step synthesis of polyurethane

A two-step synthesis of polyurethanes (prepolymers and polyurethane) is shown in Fig. 1. At first, PCL was reacted with HDI and an isocyanate-terminated prepolymer was prepared. After that 1,4-butandiol (BDO) was reacted with the prepolymer and the final product was formed. FTIR spectroscopy data indicate that the reaction has been completed.

**Fig. 2:**
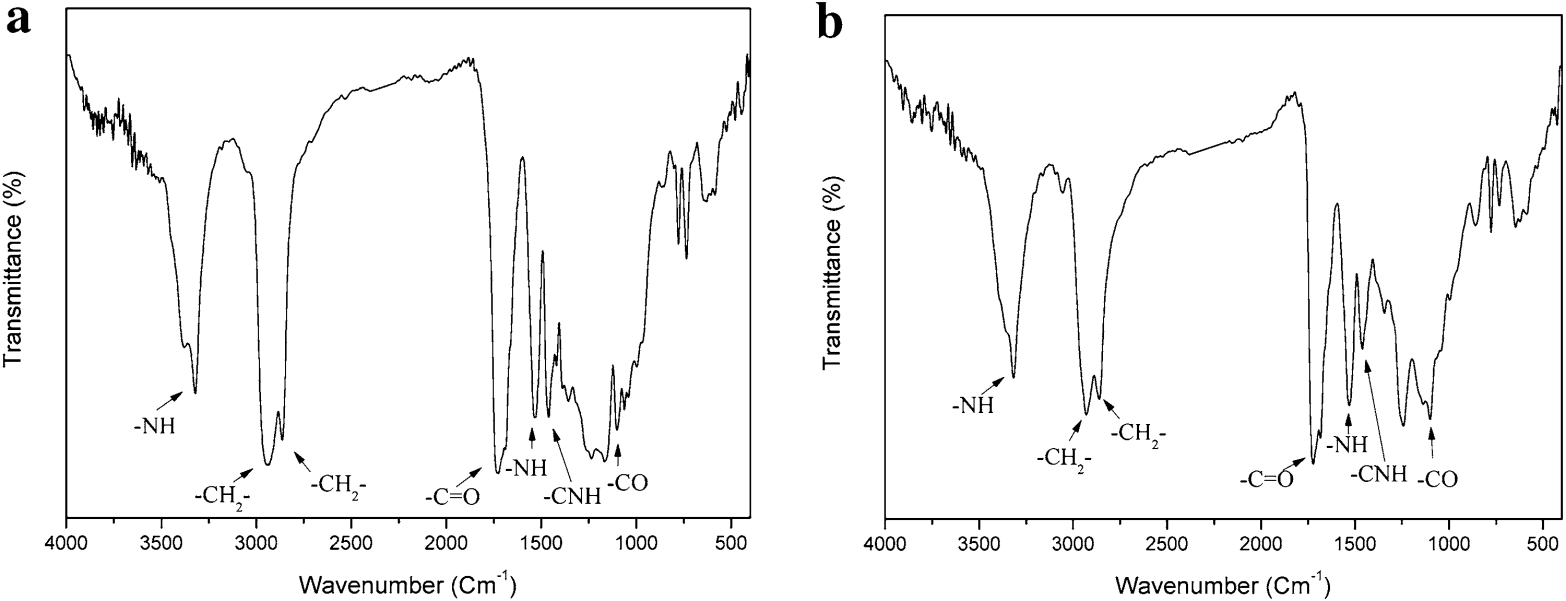
FTIR spectrum of **a** PU2000 and **b** PU530

FTIR spectra of the polymers show –NH of urethane bond absorbance at 3300–3400 and 1535 cm^−1^. The peaks, observed at 2944 and 2873 cm^−1^, are associated with the asymmetric and symmetric –CH_2_ groups. The peak at 1730 cm^−1^ is assigned to the stretching vibration of C=O groups. The peaks located at 3316, 1730, 1457 and 1100 cm^−1^ correspond to the –NH, –C=O, –CNH and –C=O absorptions and confirm the presence of –NHCOO groups in the synthesized polyurethane (Pradhan and Nayak 2012). FTIR spectrum is shown in Fig. 2 and there is no difference between the FTIR spectrum of PU2000 and PU530.

**Fig. 3:**
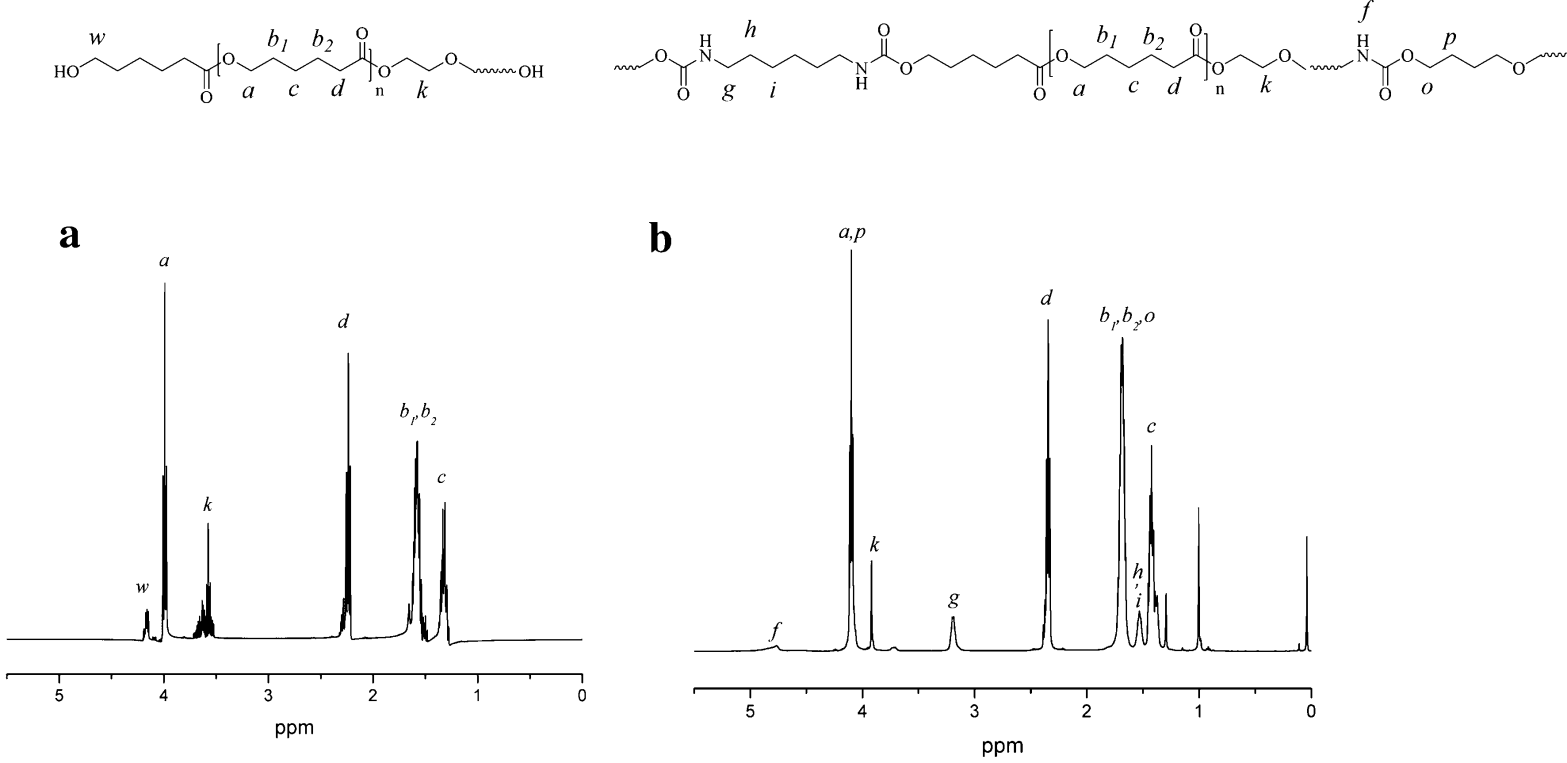
^1^H NMR spectrum of **a** PCL2000 and **b** PU2000

^1^H NMR spectrum and chemical structure of PCL2000 and PU2000 are shown in Fig. 3 and protons are marked on ^1^H NMR spectral peaks. The appearance of H_f_ in 4.8 ppm on the spectrum of PU2000 compared with that of PCL2000 indicates formation of urethane group in the product (Li et al. 2014).

**Fig. 4:**
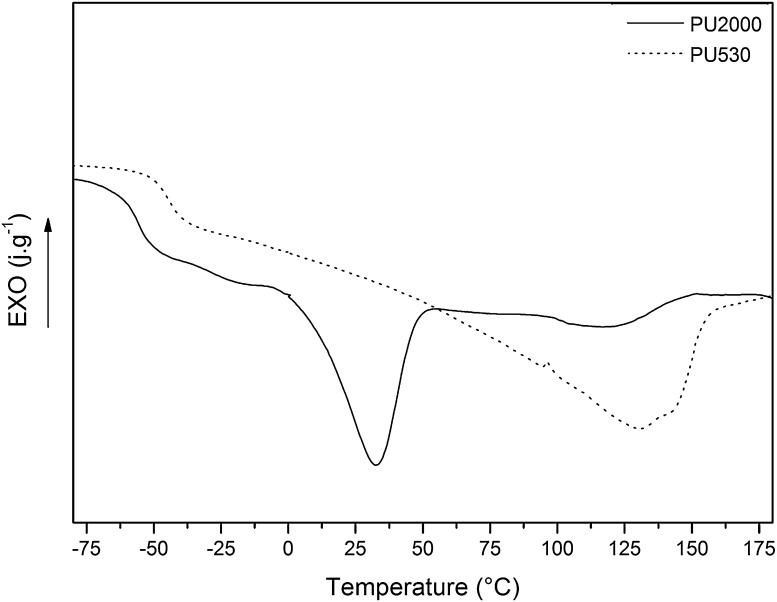
DSC curves (second heating cycle) of PU530 and PU2000

Thermal transition temperatures of two series of polyurethane are summarized in Table 2. Figure 4 shows thermograms of PU530 and PU2000. As it can be seen in Fig. 4 PU530 has shown no melting temperature for the soft segments. It seems that the hard segment formation has probably interrupted crystallization of the soft segments (Skarja and Woodhouse 1998) and soft segments remain amorphous. Glass transition temperature of PU2000 (− 55.98 °C) is lower than PU530 (− 44.78 °C) because of higher soft segment content and longer chain which could have led to facilitate chain mobility (Bajsic et al. 2000; Loh et al. 2008). The soft segment content of PU2000 is 82.5% and that of PU530 is 55.4% and hard segments melting points are observed at 130.61 °C for PU530 and 119.16 °C for PU2000. The lower enthalpy in melting point of PU2000 (− 2.97 J g^−1^) compared to PU530 (− 44.68 J g^−1^) is an indication of greater crystallinity of PU530 hard segments relative to those of PU2000.

**Table 2:** Thermal transitions of polyurethanes

Sample code	*T*_gs_ (℃)	*T*_ms_ (℃)	*T*_mh_ (℃)	Δ*H*_m_ (J g^−1^)
PU2000	− 55.98	32.50	119.16	− 2.97
PU530	− 44.78	–	130.61	− 44.68

**Fig. 5:**
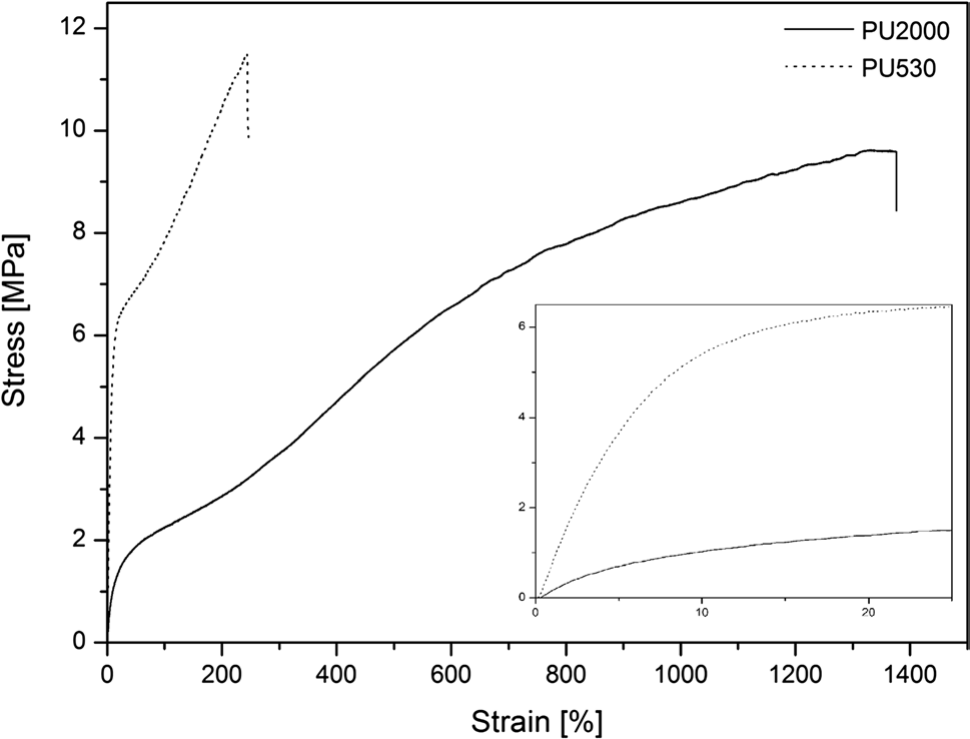
Stress–strain curves of PU2000 and PU530

Figure 5 shows stress–strain curves of the polyurethanes and modulus, tensile strength and strain-at-break extracted and reported in Table 3. Polyurethanes have hard segments, made of diisocyanate and chain extender which are responsible for the hardness of the polymer, and soft segments consisting of polyol which is responsible for the flexibility of the polymer. Also, they have microdomains formed by interactions between hard segments as hard domains and soft segments as soft domains. As it is clear hard domains act like filler through soft domains, so, polyurethane can be considered as a nanocomposite (Clough et al. 1968). Increasing hard segment leads to higher modulus and lower strain-at-break. On the other hand, increasing soft segment leads to greater flexibility followed by higher strain-at-break and lower modulus (Allport and Mohajer 1973; Bonart 1968; Clough and Schneider 1968). It can be seen that PU530 has greater modulus (4.87 MPa) and lower strain-at-break (207%) compared to PU2000 (0.75 MPa modulus and 1300% strain-at- break) because of the former higher hard segment content (44.6%). The urethane group density has been increased and, therefore, it has increased the probability of intermolecular interaction which has led to greater resistance against deformation. Meanwhile, PU2000 has greater flexibility because of its higher soft segment content (82.5%).

**Table 3:** Mechanical properties of polyurethanes

Sample code	Modulus (MPa)	Tensile strength (MPa)	Strain at break (%)
PU2000	0.75 ± 0.07	9.80 ± 0.20	1300 ± 30
PU530	4.87 ± 0.18	11.74 ± 0.36	207 ± 31

**Fig. 6:**
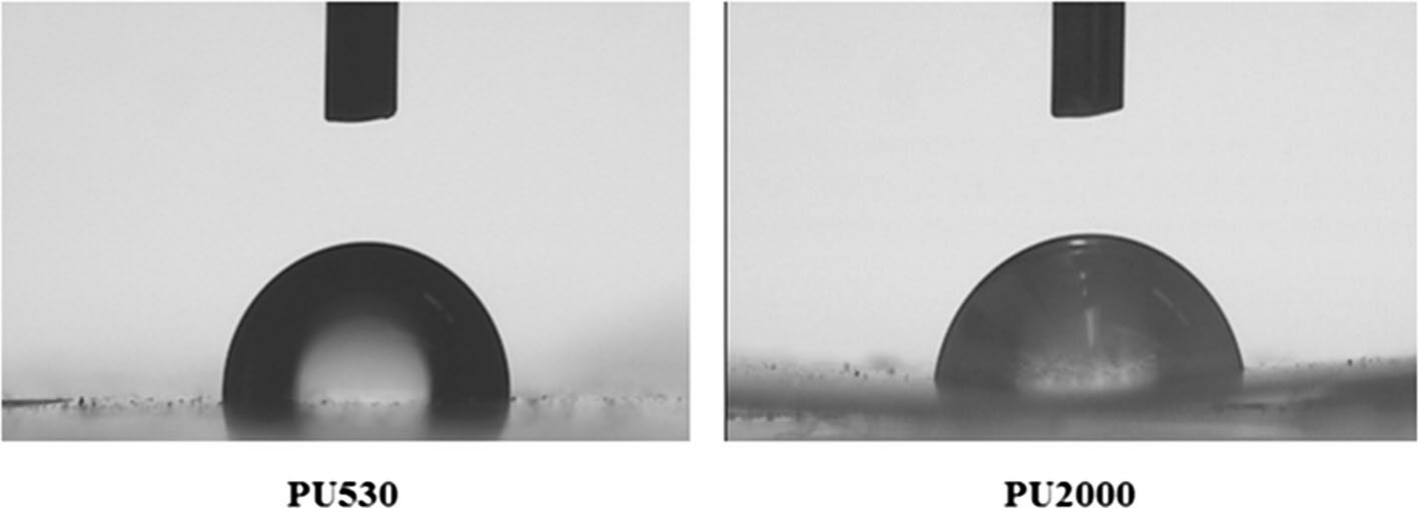
Water contact angle of PU2000 and PU530

Surface hydrophilicity of the polyurethanes was analyzed by static water contact angle measurements for which the results are shown in Fig. 6. Contact angle and water uptake data are reported in Table 4. As can be seen the increase in molecular weight of polycaprolactone has increased hydrophilicity as shown by lower water contact angle (85.8º for PU2000 and 97º for PU530) and small increase in water uptake (3.4% for PU2000 and 2.1% for PU530).

**Table 4:** Water contact angle and water uptake data of PU530 and PU2000

Sample code	Contact angle (°)	Water uptake (°)
PU2000	85.8 ± 2.6	3.4 ± 0.8
PU530	97.0 ± 1.2	2.1 ± 0.5

Water uptake of PU2000 and PU530 shows no significant difference as reported by Skrja et al. (Skarja and Woodhouse 1998). A little higher water uptake of PU2000 may be because of more hydrophobicity of PCL530 and more hydrophilic surface of PU2000.

## Curcumin-loaded electrospun nanofibers

**Fig. 7:**
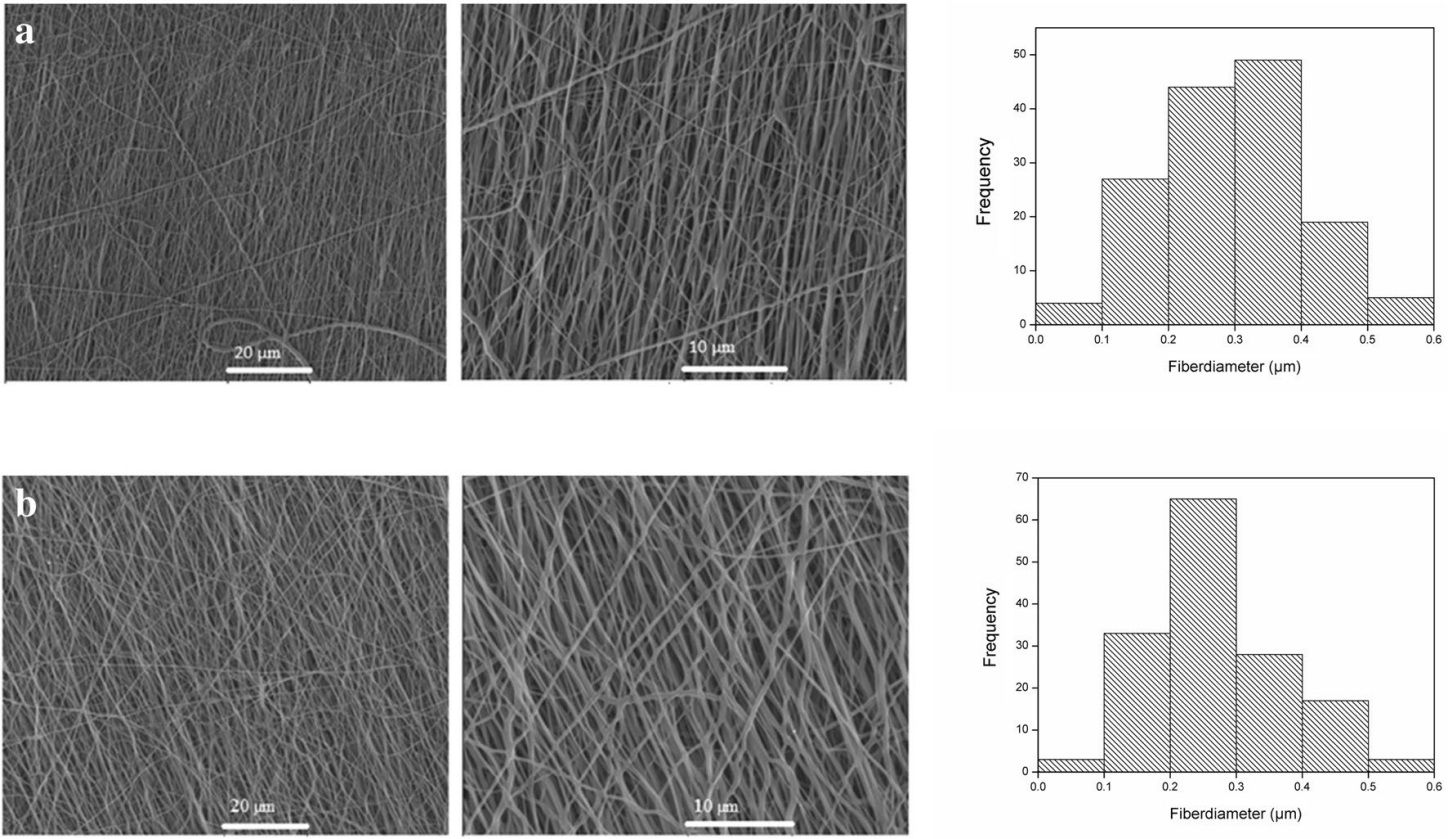
SEM images of PU530: **a** 0% curcumin loaded **b** 5% curcumin loaded with related histogram

**Fig. 8:**
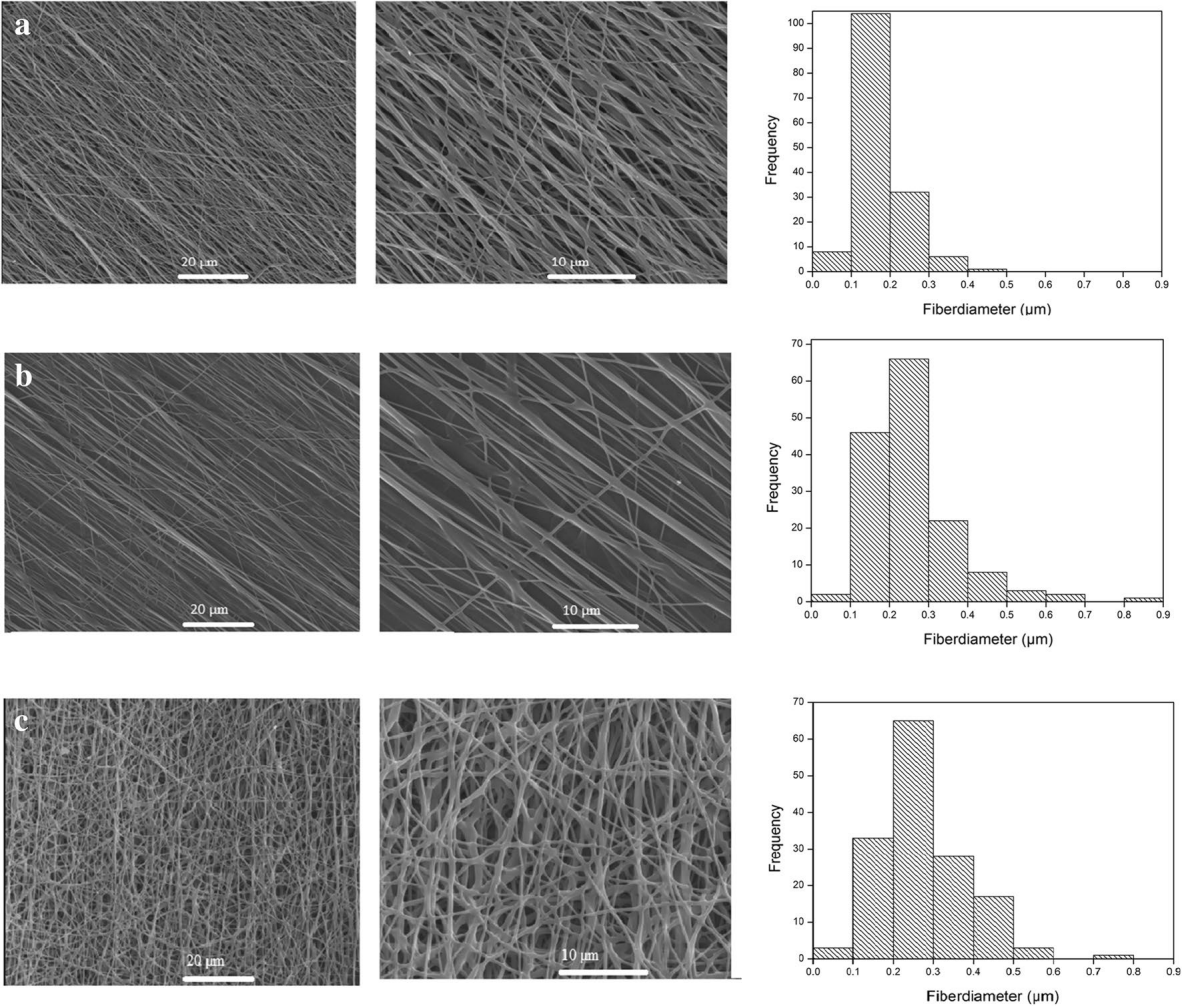
SEM images of PU2000: **a** 0% curcumin loaded **b** 5% curcumin loaded **c** 10% curcumin loaded with related histogram

**Fig. 9:**
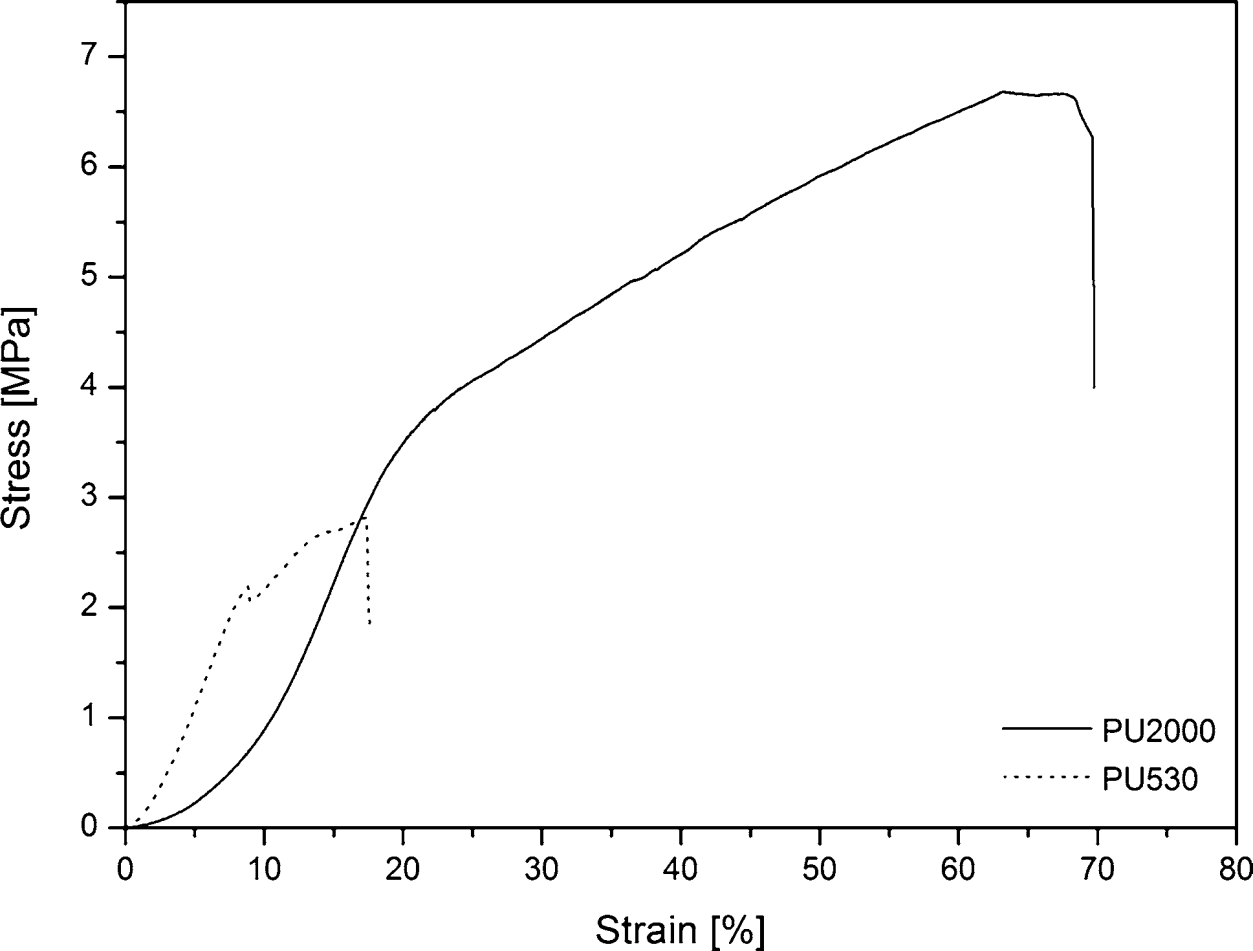
Stress–strain curves of the electrospun nanofibers of PU2000 and PU530

To fabricate PU nanofibrous substrate loaded curcumin, electrospinning method has been used because of its many advantages such as large surface area. Morphology of the nanofibers was investigated by SEM and is illustrated in Figs. 7 and 8. As can be seen in these figures, surface of the nanofiber is smooth and no bead is observed. There is no crystal of curcumin observed which means curcumin has been completely incorporated into the fibers (Fu et al. 2014). The average fiber’s diameter is shown in Table 5. Average diameter is increasing by incorporation of curcumin in nanofiber. Nanofiber diameters of PU530 and PU2000 are summarized in Table 5. Incorporation and increases of curcumin in nanofibers have led to higher average fiber diameter, as reported by some researchers on curcumin incorporated into other polymers (Chen et al. 2010; Suwantong et al. 2007). Increases in average fiber diameter can be disregarded by the processing condition (e.g., polymer concentration, voltage, tip-to-collector distance and flow rate) which all lead to constant fiber diameter. It seems that the incorporation of drug into polymer nanofibers may be the only parameter which is responsible in fibers of greater diameter. Stress–strain curves are shown in Fig. 9 and the mechanical properties are reported in Table 6. Mechanical properties of the 5% curcumin loaded electrospun mats follow the same trend as mechanical behaviors of the copolymer films. As it was mentioned above, PU530 has higher modulus because of higher hard segment content and PU2000 has more flexibility because of higher soft segment content. In this study we have used 5% curcumin loaded electrospun mats which have led to higher elongation and higher strength-at-break and lower modulus compared with electrospun mates without curcumin (Ranjbar-Mohammadi and Bahrami 2016).

**Table 5:** Average fiber diameter of electrospun nanofiber polyuerthanes

Sample code	Curcumin content (%)	Average fiber diameter (nm)
PU2000	0	172 ± 64
	5	256 ± 110
	10	274 ± 107
PU530	0	200 ± 80
	5	284 ± 112

**Table 6:** Mechanical properties of electrospun nanofibers mats of polyurethanes

Sample code	Modulus (MPa)	Tensile strength (MPa)	Strain at break (%)
PU2000	5.96 ± 0.13	6.90 ± 0.30	65 ± 8
PU530	18.50 ± 2.12	2.65 ± 0.21	18 ± 1

To acquire bead free electrospun fibers which is the aim of this study, curcumin loading capacity has been restricted. The highest curcumin loading for PU530 is found 5% because it cannot be dissolved at higher amount, and, therefore, 10% curcumin has just been loaded in PU2000 because of lower polymer solution concentration.

### Curcumin release study

**Fig. 10:**
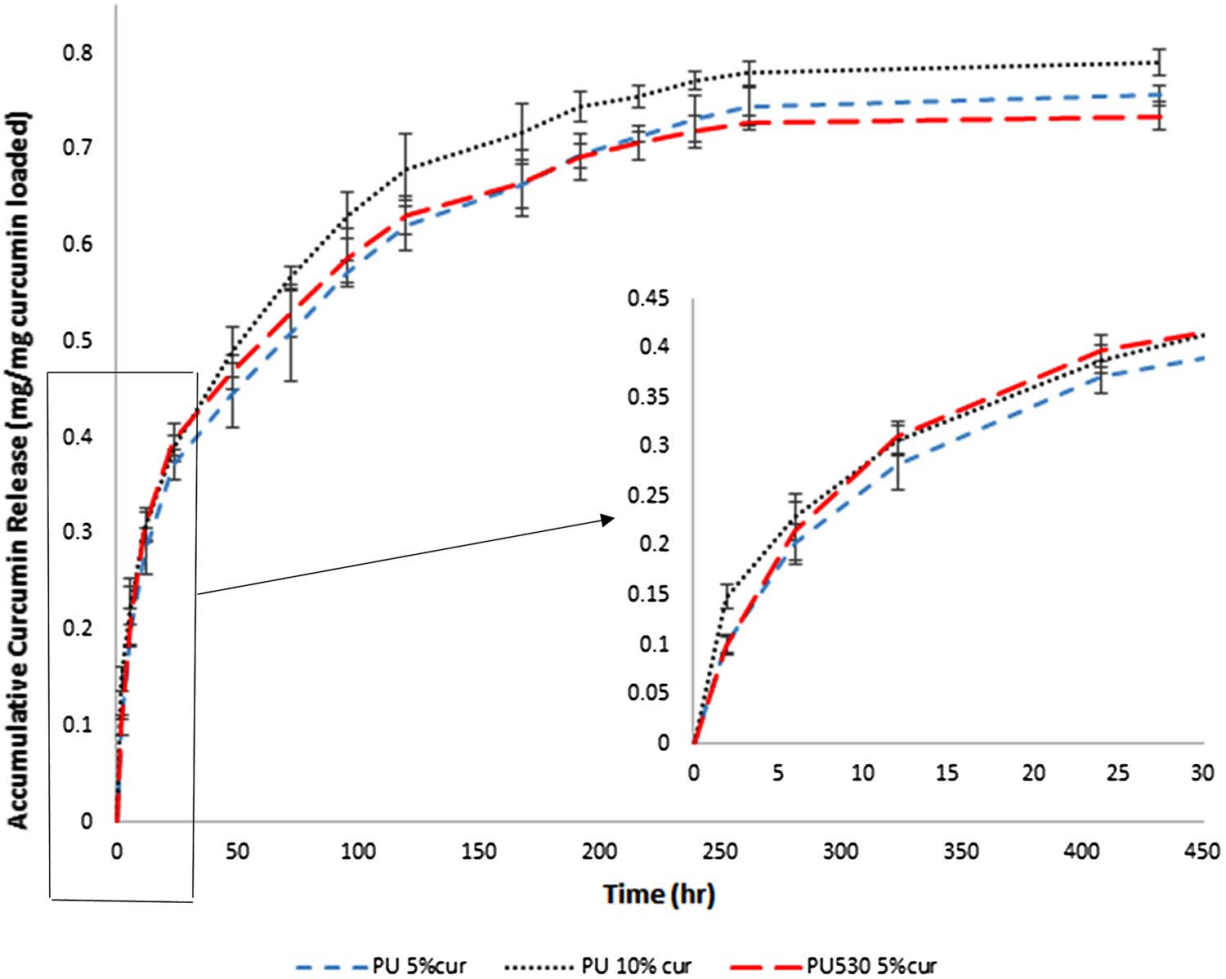
Curcumin release study in release medium (70:30 water:ethanol)

Curcumin release curve is plotted by the accumulative curcumin release (mg released curcumin divided by initial curcumin loaded) versus time and is shown in Fig. 10. Curcumin release in both polyurethanes follows the same pattern in which at first they show a burst release (for 24 h) because of dissolution of curcumin accumulated onto the surface followed by the sustained release of the interior curcumin over 11 days. As it is obvious in PU2000 with more hydrophilic surface, the burst release is lower than PU530, because curcumin prefer**s** to remain in hydrophobic segments of the polymer (Sampath et al. 2014). PU2000 with 10% curcumin loading showed more burst release than PU2000 (loaded by 5%). It is because PU2000 surface has contained more curcumin and the maximum amount of release of PU2000 with 10% (~ 80%) is still higher than the polyurethanes loaded by 5% (~ 72%), as it is indicated in Fig. 10.

### In vitro anti-bacterial properties

**Fig. 11:**
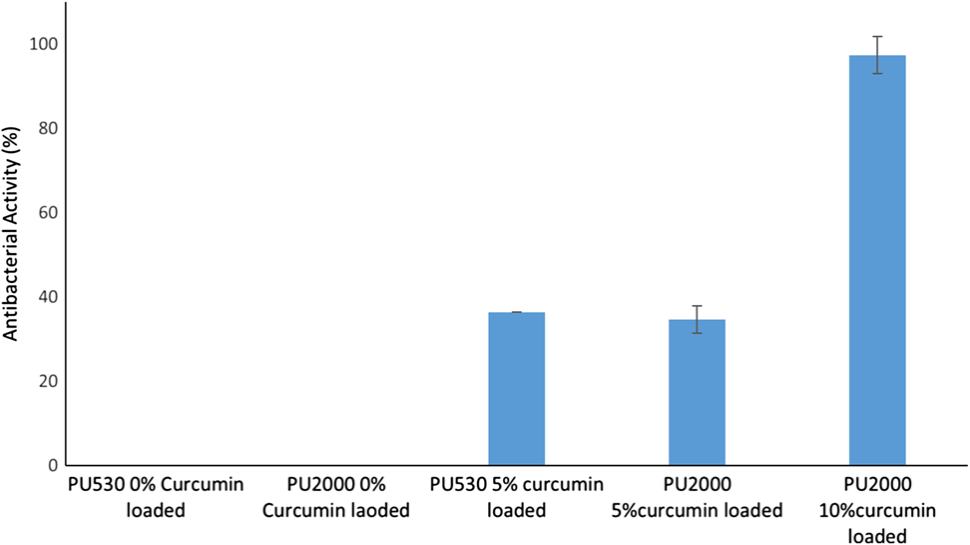
Antibacterial activity of the electrospun curcumin-loaded polyurethanes

The results of anti-bacterial properties of curcumin-loaded polyurethane mats against 10^5^ CFU ml^−1^ of E.coli are shown in Fig. 11. The electospun mats, of each 50 mg, were placed into 5 mL bacterial solution. Each mat was loaded by 5% curcumin and contained ~ 2.5 mg curcumin and the PU 2000 electrospun mat which was loaded by 10% curcumin contained approximately 5 mg curcumin. In this research, a turbidimetric assay was used to study the anti-bacterial activity of the curcumin-loaded electrospun polyurethane nanofibers (Aulton 2002). Therefore, the maximum concentration of curcumin in solution for 5% curcumin-loaded PU530 and PU2000 and 10% curcumin-loaded PU2000 would be ~ 500, 500 and 1000 µg mL^−1^, respectively. The minimum inhibitory concentration (MIC) of curcumin against Escherichia coli ATCC:25922 is 163 µg mL^−1^ (Gunes et al. 2013). As regards the release process by ~ 42% in 48 h there were 48 and 45% released from 5 to 10% curcumin-loaded PU2000 and 5% curcumin-loaded PU530 were obtained which implied approximately 210 and 480 µg mL^−1^ curcumin release from 5 to 10% curcumin-loaded mats, respectively. It could be concluded that the anti-bacterial efficiency of 10% loaded PU2000 showed higher anti-bacterial efficiency by elimination of most bacteria (97% anti-bacterial activity).

## Conclusion

In this study two series of polyurethanes were synthesized in a two-step polymerization based on PCL (*M*_n_ 530 and 2000 Da), HDI and BDO. FTIR and ^1^H NMRspectroscopy characterization confirmed completion of polyurethanes synthesis. Thermal properties of the polyurethanes were studied by DSC and the results showed that soft segments of PU530 are amorphous and hard segments of this polymer are crystalline. Hydrophilicities of the synthesized polymers were studied and the results showed that PU2000 is more hydrophilic in nature than PU530. Curcumin was loaded in the polyurethanes by electrospinning process in different contents. The release rate of curcumin from polyurethane matrices as well as anti-bacterial activity of the mats was investigated and the results demonstrated that PU2000, because of good mechanical and anti-bacterial properties, is a good candidate for wound dressing applications.
